# Spin and Orbital States in Triclinic Ilmenite-Type CuVO_3_

**DOI:** 10.1021/acsomega.5c13303

**Published:** 2026-03-31

**Authors:** Hajime Yamamoto, Keigo Ochi, Takuya Aoyama, Kenji Ishii, Daiju Matsumura, Takuya Tsuji, Kenya Ohgushi, Tadashi Abukawa

**Affiliations:** † Institute of Multidisciplinary Research for Advanced Materials, 13101 Tohoku University, 2-1-1 Katahira, Aoba-ku, Sendai, Miyagi 980-8577, Japan; ‡ Department of Physics, Graduate School of Science, 13101 Tohoku University, 6-3 Aramaki-Aoba, Aoba-ku, Sendai, Miyagi 980-8578, Japan; § Kansai Institute for Photon Science, National Institutes for Quantum Science and Technology (QST), 1-1-1 Kouto, Sayo-cho, Sayo-gun, Hyogo 679-5198, Japan; ∥ Japan Atomic Energy Agency (JAEA), 1-1-1 Kouto, Sayo-cho, Sayo-gun, Hyogo 679-5198, Japan; ⊥ International Center for Synchrotron Radiation Innovation Smart, 13101 Tohoku University, 2-1-1 Katahira, Aoba-ku, Sendai, Miyagi 980-8577, Japan

## Abstract

Ilmenite-type CuVO_3_ is a promising compound for exploring novel spin and orbital states, as Cu^2+^ (3d^9^) and V^4+^ (3d^1^) ions each form a honeycomb lattice. In this study, we synthesized triclinic ilmenite-type CuVO_3_ using a high-pressure and high-temperature method and investigated its spin and orbital states through synchrotron X-ray diffraction and measurements of electrical resistivity, magnetic susceptibility, and specific heat. The Cu^2+^ ions exhibit the Jahn–Teller effect, leading to the triclinic distortion. This Jahn–Teller distortion hinders the formation of static V–V dimers, in contrast to other ilmenite-type vanadium oxides in which V–V dimerization is observed. These findings highlight triclinic ilmenite-type CuVO_3_ as a fascinating system in which each cation exhibits characteristic spin and orbital states.

## Graphical abstract

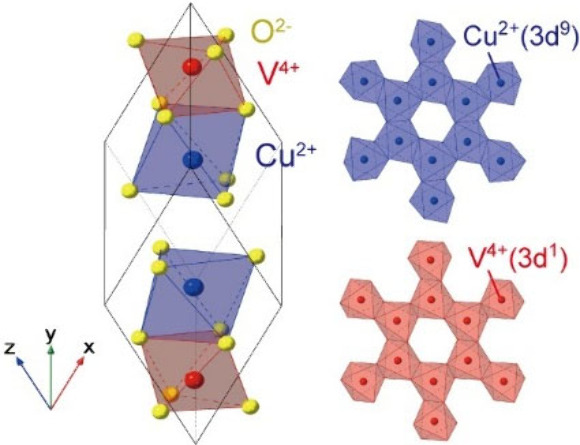

## 1 Introduction

Divalent Cu and tetravalent V ions exhibit 3d^9^ and 3d^1^ electronic configurations, respectively. Both ions have a total spin of 1/2. Because of the orbital degrees of freedom associated with their e_g_ or t_2g_ orbitals, honeycomb lattice compounds containing Cu^2+^ and V^4+^ are known to exhibit novel phenomena. For example, a spin–orbital liquid state is realized in a Cu^2+^-honeycomb lattice system, Ba_3_CuSb_2_O_9_, where the spins and orbitals remain unfrozen even at low temperatures.^1,2^ In ilmenite-type vanadium oxides (A^2+^V^4+^O_3_) with honeycomb lattices of V^4+^, vanadium ions undergo dimerization to form covalent bonds between the 3d orbitals.^3,4^

Ilmenite-type CuVO_3_ is a promising compound for exploring novel spin and orbital states, as Cu^2+^ (3d^9^) and V^4+^ (3d^1^) ions each form a honeycomb lattice. The CuO_6_ and VO_6_ octahedra are linked by edge-sharing to form the honeycomb lattices. Ilmenite-type CuVO_3_ can be synthesized at high pressures and temperatures; however, the two polymorphs (triclinic and rhombohedral ilmenite-type phases) have been reported.^5–7^ The occurrences of these two phases depend on the reactants and the synthesis conditions owing to their off-stoichiometry.^5^ The triclinic compound is stoichiometric. Both phases exhibit a gold color, and the triclinic one exhibits a small magnetic moment and low electrical resistivity. Previous crystallographic studies have revealed considerable V 3d–V 3d overlaps and interactions in the honeycomb lattice of the triclinic phase, resulting in itinerancy and reduced magnetic moments.^6^ The valence states of Cu^2+^ and V^4+^ were predicted from the crystal structure; however, the origin of the triclinic distortion remains unclear.

Triclinic distortion is a characteristic feature of ilmenite-type vanadium compounds, including MgVO_3_, MnVO_3_, CoVO_3_, NiVO_3_, and ZnVO_3_. This distortion originates from V–V dimerization.^3,4,8–12^ Except for ZnVO_3_, these compounds exhibit reversible structural transition from low-temperature triclinic to high-temperature rhombohedral phase around 450–550 K. Below the transition temperature, covalent bonds form between adjacent V ions on the honeycomb lattice, resulting in V–V dimerization accompanied by metal-to-insulator and magnetic-to-nonmagnetic transitions.^3,4^ The V–V dimers are arranged in a ladder-like pattern within the honeycomb lattice. V–V dimerization occurs by selecting one of the three V–V directions (3d_*xy*_, 3d_*yz*_, and 3d_*xz*_ orbitals), which breaks the trigonal symmetry.^3^ In CuVO_3_, the V–V dimerization does not occur; however, V 3d–V 3d interactions and overlaps may exist along several directions.^6^

In this study, we focused on triclinic CuVO_3_, which features both Cu^2+^ and V^4+^ honeycomb lattice systems, to explore the novel quantum phenomena at low temperatures. Triclinic CuVO_3_ samples were synthesized at high pressure and temperature (8 GPa and 800 °C, respectively). X-ray absorption spectroscopy (XAS) confirmed that the valence state was predominantly Cu^2+^V^4+^O_3_. The obtained sample exhibits insulating properties and shows no magnetic phase transitions down to 2 K. Crystal structure refinement using synchrotron X-ray diffraction (SXRD) data revealed the Jahn–Teller distortion of the CuO_6_ octahedron and the absence of V–V dimers. The Jahn–Teller effect of Cu^2+^ likely prevents the formation of the V–V dimers in CuVO_3_. Triclinic ilmenite-type CuVO_3_ is an unusual compound in which different elements adopt distinct orbital states.

## 2 Experimental Section

CuVO_3_ samples were synthesized at 8 GPa and 1073 K for 1 h using a Walker-type multianvil apparatus.^13^ The starting mixture was CuO + 0.6V_2_O_5_ + 0.4V_2_O_3_, and the composition ratio was CuVO_3.1_. The obtained samples were ground in an agate mortar. SXRD measurements were performed at a temperature range of 100–600 K using BL02B2, SPring-8 with a wavelength of λ = 0.4140 Å (*E* = 29.95 eV). The sample was sealed in a glass capillary with a diameter of 0.2 mm in air, and the temperature was controlled using a nitrogen gas flow system. Rietveld analysis was performed using RIETAN-FP.^14^ XAS was performed using the transmission method at BL14B1, SPring-8 to measure the K-edges of Cu and V. The electrical resistivity of the samples was measured using the four-probe method. The temperature dependence of the magnetic susceptibility and the magnetic-field dependence of the magnetization of the CuVO_3_ samples were measured using a superconducting quantum interference device magnetometer (MPMS-XL, Quantum Design). The specific heat was measured using a physical-property measurement system (Quantum Design).

## 3 Results and Discussion

Triclinic ilmenite-type CuVO_3_ was successfully synthesized at high pressure and temperature. Figure 1a shows the SXRD pattern at 100 K and the Rietveld analysis results for the sample. Small amounts of unknown impurities were observed (marked with asterisk in Figure 1a). CuVO_3_ crystallized in the triclinic *P*1̅ space group. Tables S1–S4 list the lattice and structural parameters at 100 and 300 K. There was no significant site-mixing between the Cu and V atoms; thus, their occupancies were fixed at 1. The temperature factor *B* of the oxygen atoms remained within a reasonable range when the occupancy was fixed at 1, indicating the absence of oxygen deficiency. As evident by the decomposition of CuO into Cu_2_O at high temperatures, Cu^2+^ tends to be reduced to Cu^+^ during the synthesis conditions. Therefore, stoichiometric compounds were successfully obtained under oxygen-rich conditions. Figure 1b shows the refined crystal structure of the sample at 100 K. The structure is similar to that reported in a previous study.^6^ Two of the three V–V distances in the V honeycomb lattice were relatively short (2.893(5) and 2.910(6) Å), which allowed for an overlap between the 3d orbitals of the adjacent V ions.^15^ SXRD was also performed in the temperature range of 100–600 K (Figure S1). The triclinic structure was preserved up to 600 K. At temperatures above 500 K, the diffraction peaks became broad, and the unit cell volume increased (Figure S2), indicating the occurrence of oxygen vacancies in the structure due to heating. This peak broadening was irreversible upon cooling. Figure 2 shows the X-ray absorption spectra of the Cu and V K-edges of CuVO_3_ and the reference samples at 300 K. The valence state was predominantly Cu^2+^V^4+^O_3_, as estimated from the energy positions of the absorption edges and pre-edge peaks.

**Figure 1:**
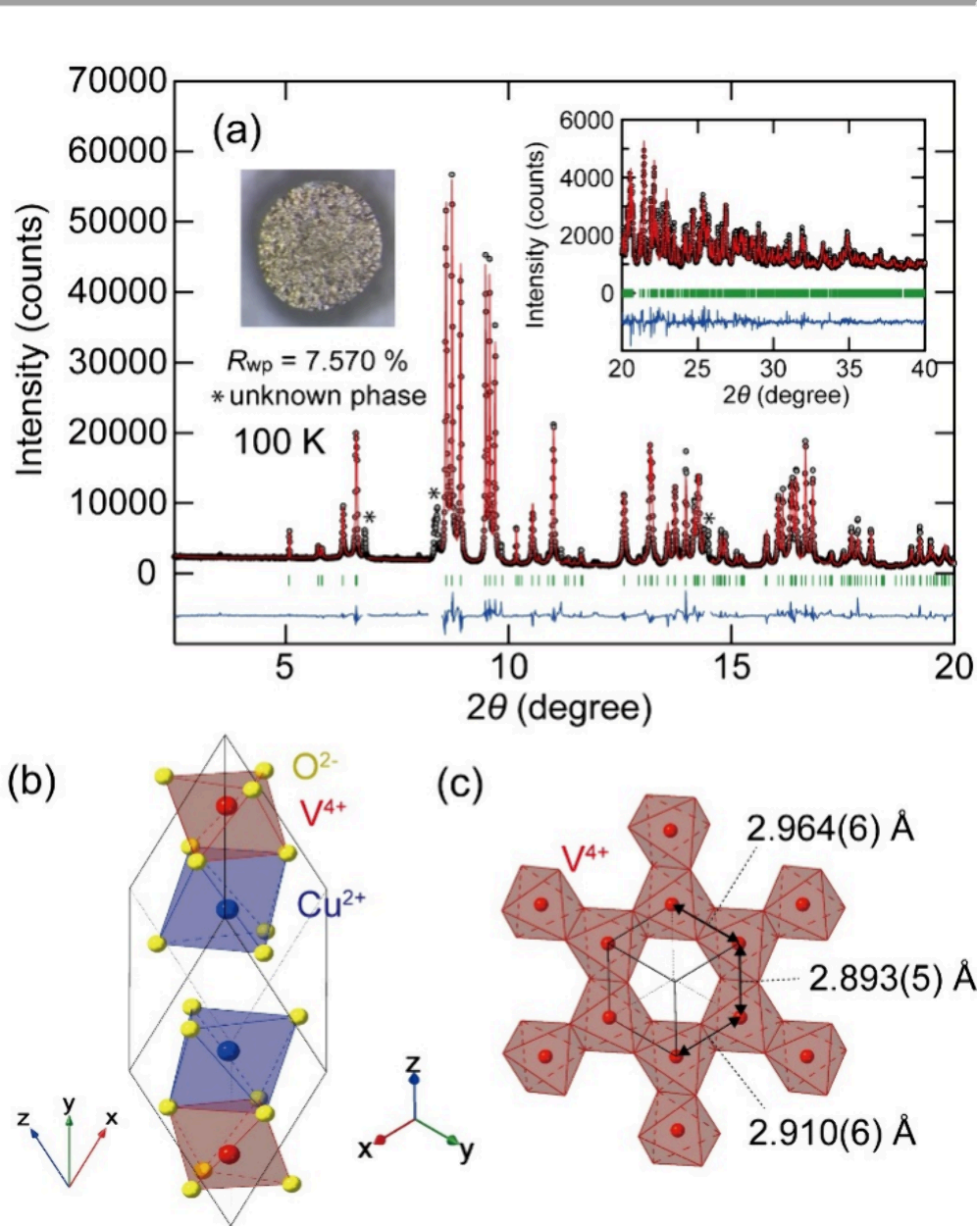
(a) Synchrotron X-ray diffraction (SXRD) pattern of triclinic ilmenite-type CuVO_3_ at 100 K and the Rietveld refinement results. The inset shows the obtained sample. (b) Refined crystal structure. (c) V^4+^ honeycomb lattice and V–V distances within the honeycomb lattice.

**Figure 2:**
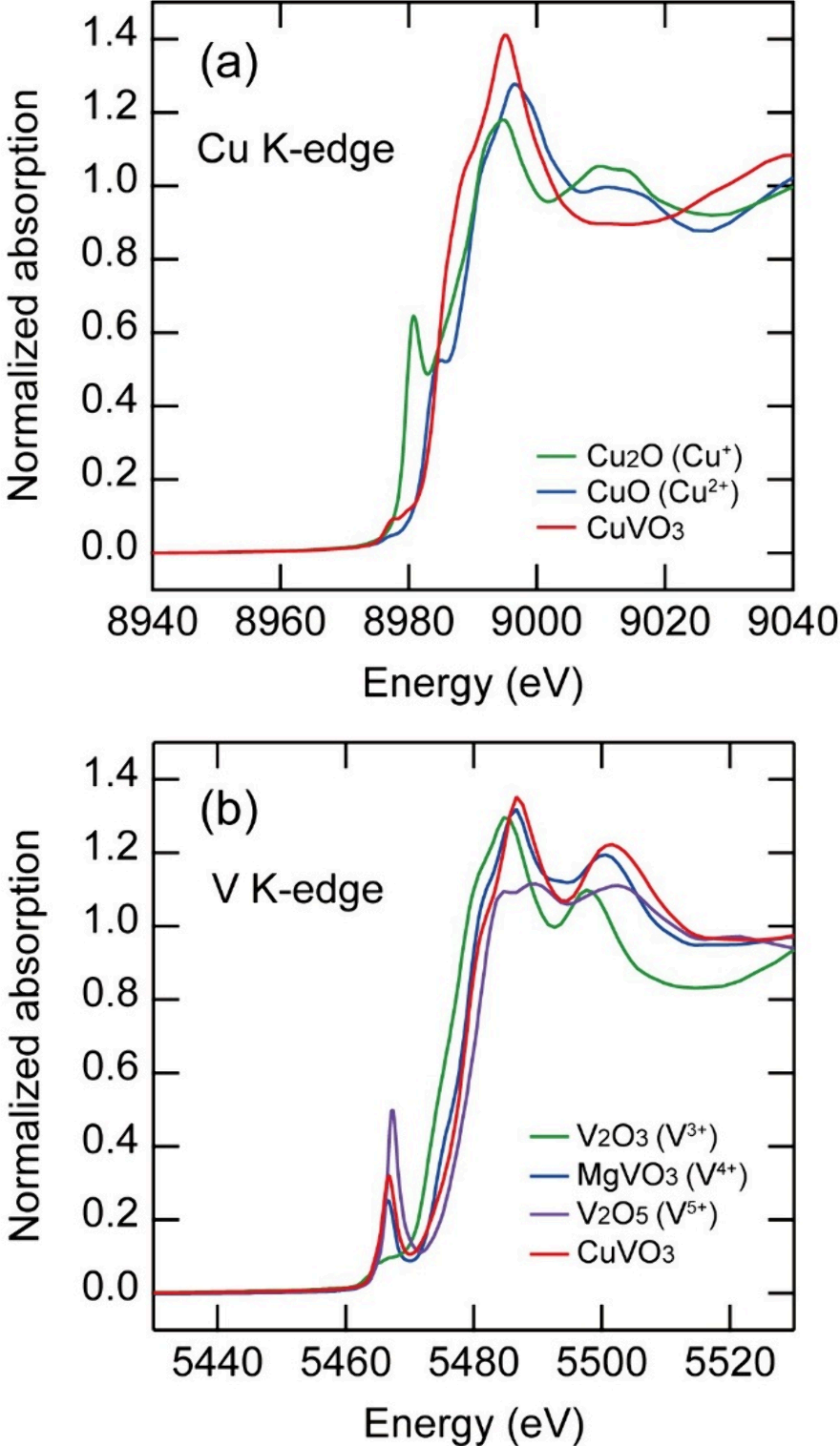
(a) X-ray absorption spectra of Cu K-edges of CuVO_3_, CuO, and Cu_2_O at 300 K. (b) X-ray absorption spectra of V K-edges of CuVO_3_, MgVO_3_, V_2_O_3_, and V_2_O_5_ at 300 K.

Figure 3a shows the temperature dependence of the electrical resistivity of CuVO_3_. CuVO_3_ exhibited an insulating behavior below 300 K. The electrical resistivity at room temperature was ∼1 Ω·cm. The activation energy *E*_g_ was 0.1019(1) eV in the temperature range of 196–295.6 K (Figure 3b). Figure 3c shows the temperature dependence of the magnetic susceptibility of CuVO_3_. The curve shows Curie–Weiss-like behavior, and no anomaly related to magnetic transitions is observed at temperatures down to 2 K. The magnetic susceptibility at 150–300 K was well reproduced by the Curie–Weiss law with the addition of a temperature-independent term χ_0_. The Curie constant was *C* = 0.0844(2) emu·K/mol, the Weiss temperature was θ = 17.5(2) K, and the temperature-independent term was χ_0_ = 8.21(5) × 10^–5^ emu/mol. The low Curie constant might be attributed to the partial itinerancy of 3d electrons or hybridization between Cu and V 3d orbitals. Such a low Curie constant was also reported in a previous study.^5^ A positive Weiss temperature suggests ferromagnetic interactions; however, this interpretation is not conclusive because the magnetic moment is small and the temperature-independent contribution is relatively large. A magnetization curve in line with the Brillouin function was observed at 2 K, indicating that the compound exhibits paramagnetic behavior at such low temperatures.

**Figure 3:**
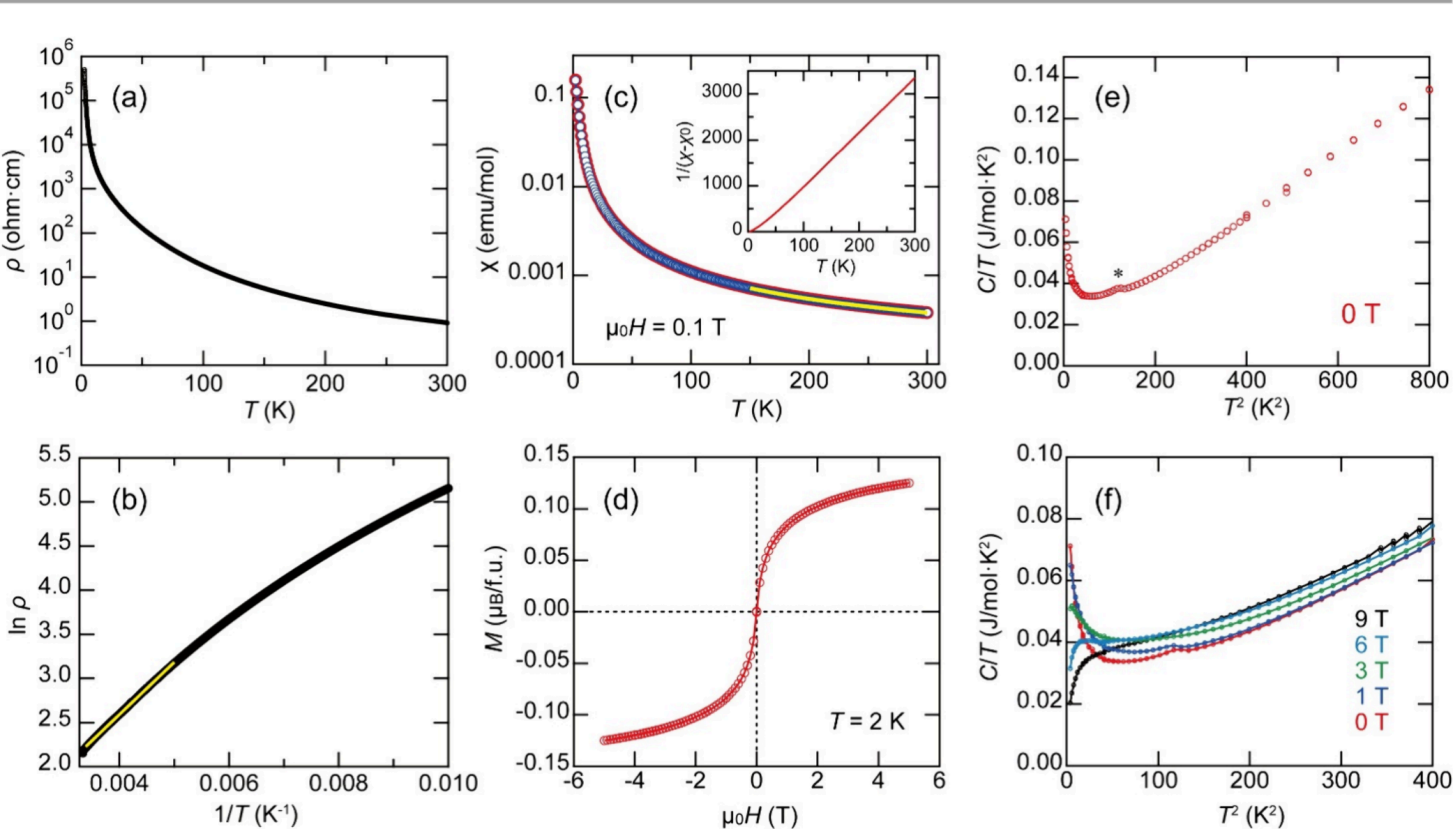
(a) Temperature dependence of the electrical resistivity of ilmenite-type CuVO_3_. (b) Arrhenius plot of the resistivity data and the fitting result (yellow line). (c) Temperature dependence of the magnetic susceptibility of CuVO_3_ and the Curie–Weiss fitting curve (yellow curve). The red and blue lines indicate the field-cooling (FC) and zero-field-cooling processes, respectively. The inset shows the reciprocal plot of the FC process. (d) Magnetic-field dependence of magnetization of CuVO_3_. (e) Temperature dependence of the specific heat (*C*/*T* vs *T* ^2^ plot) of CuVO_3_ at 0 T. (f) Temperature dependence of the specific heat (*C*/*T* vs *T* ^2^ plot) of CuVO_3_ at various magnetic fields.

Figure 3e shows the specific heat (*C*/*T* vs *T* ^2^ plot) at 0 T. An upturn in *C*/*T* was observed toward the lowest temperatures, and a small peak indicating a phase transition appeared at 11 K (marked with an asterisk in Figure 3f). Since the anomalies are present up to around 15 K, an accurate estimation of the lattice contribution from these data is not feasible. Figure 3f shows the magnetic-field dependence of the specific heat. The broad anomaly, the upturn in *C*/*T* at 0 T, was shifted toward higher temperatures with increasing magnetic fields, indicating the development of a short-range magnetic order. The change in magnetic entropy was much smaller than the expected value, indicating that the spins are fluctuating even at 2 K. The small peak becomes more smeared under an applied magnetic field, indicating that it originates from a magnetic phase transition. However, since the peak is very small, it is unlikely to be associated with long-range magnetic ordering of CuVO_3_ and is instead presumed to arise from a local spin ordering of Cu^2+^ (to be discussed later).

To discuss the electronic orbital state of the Cu^2+^ ions, we first consider the coordination environment. The undistorted rhombohedral ilmenite-type structure has three long and three short metal–O bonds in the oxygen octahedron. As shown in Figure 4a, the CuO_6_ octahedron was deformed to elongate along the apical direction, which can be attributed to the Jahn–Teller effect of Cu^2+^(3d^9^). This shows that the triclinic structure results from the Jahn–Teller distortion rather than V–V dimerization. Cu^2+^ has three e_g_ electrons, and two of them occupy the 3d_*z* ^2^_ orbital stabilized in the coordination geometry. Figure 4b shows the arrangement of the half-occupied 3d_*x* ^2^–*y* ^2^_ orbitals. Considering their orbital overlaps, superexchange interaction paths are possible within the Cu pairs circled in red, as shown in Figure 4b. The Cu–O–Cu angle of 89.18° indicates ferromagnetic interactions.^16,17^ An isolated ferromagnetic spin dimer interaction is established in the Cu honeycomb lattice, likely because of the weak interdimer interaction due to the absence of orbital overlaps. This is consistent with the experimental results, including the positive Weiss temperature and paramagnetic behavior persisting down to 2 K. The magnetic susceptibility deviated from the Curie–Weiss line at low temperatures, indicating the presence of weak antiferromagnetic interactions between the ferromagnetic spin dimers. Such isolated ferromagnetic spin dimers often exhibit no magnetic transitions even at low temperatures.^18^ The specific heat peak observed at 11 K might be related to the local spin ordering within the Cu spin dimers. Such magnetic ordering is likely subtle and therefore could not be detected by magnetic susceptibility measurements. From the present results, we propose that ferromagnetic dimer interaction of Cu spins occurs in CuVO_3_, and these spins fluctuate even at low temperature. Further studies, including neutron inelastic scattering and theoretical calculations, are necessary to reveal the interaction conclusively.

**Figure 4:**
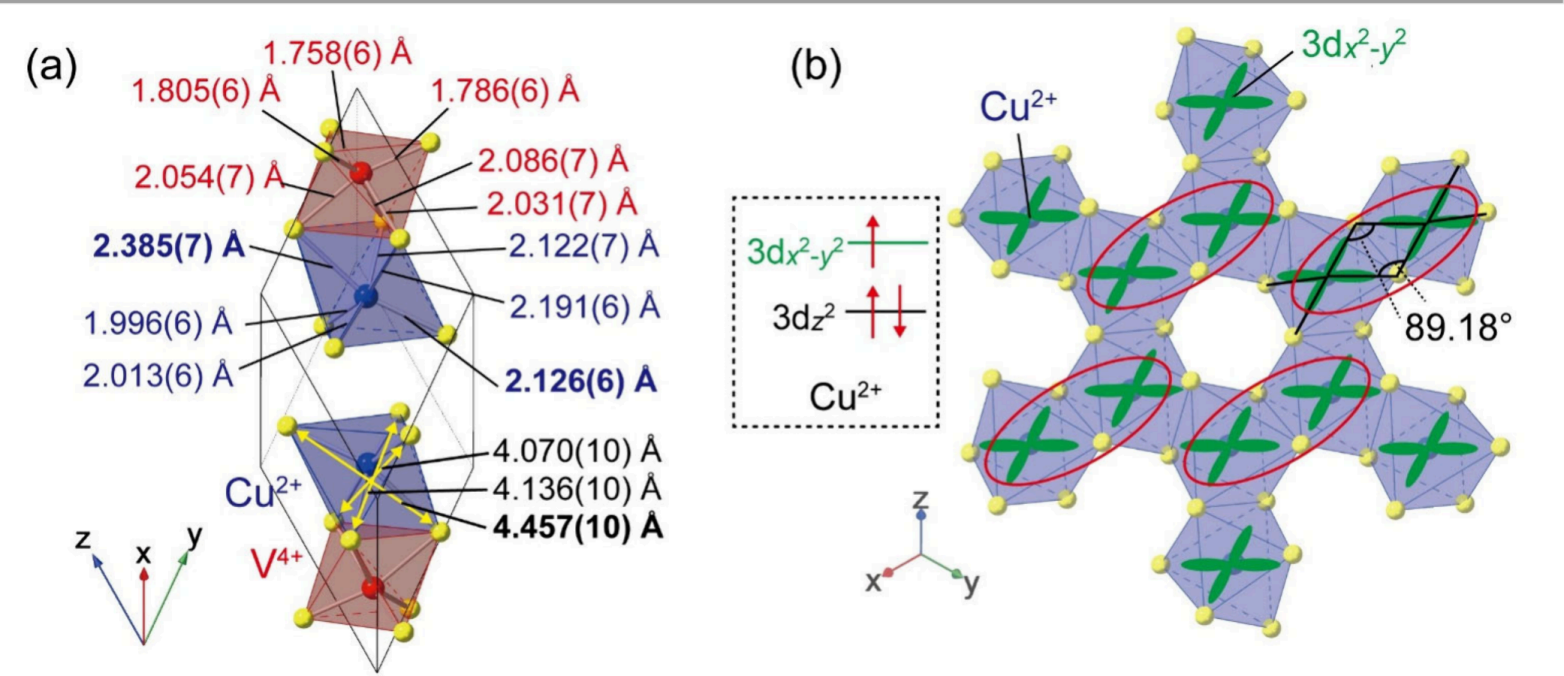
(a) Cu–O and V–O bond lengths and distances between the vertices of the CuO_6_ octahedron of ilmenite-type CuVO_3_ at 100 K. (b) Cu^2+^ honeycomb lattice and arrangement of 3d_*x* ^2^–*y* ^2^_ orbitals. The inset shows the electronic configuration of Cu^2+^.

We now discuss the orbital state of V^4+^ ions. In the VO_6_ octahedron, the lengths of three long and three short V–O bonds are similar within each group (Figure 4a). Unlike the CuO_6_ octahedron, the VO_6_ octahedron shows a small deformation from the rhombohedral structure. The tetravalent V ion has one 3d electron, which occupies the almost degenerate 3d_*xy*_, 3d_*yz*_, and 3d_*xz*_ orbitals. In other ilmenite-type vanadium oxides, one of the three orbitals is selected to form the V–V bonds. However, this selection does not occur in CuVO_3_, which may result in the formation of several molecular orbitals with similar energy levels. The absence of the static V–V dimer state may be attributed to the Jahn–Teller effect of Cu^2+^. Both Cu^2+^ and V^4+^ ions induce triclinic distortion in the ilmenite-type structure; however, these effects do not occur cooperatively but competitively. The Jahn–Teller distortion is dominant, preventing the formation of V–V dimers in CuVO_3_. In contrast, for ilmenite-type ZnVO_3_, Zn^2+^ favors the triclinic structure; however, it further stabilizes the V–V dimer structure.^12^ The Zn ions prefer tetrahedral coordination, whereas the Cu ions prefer square-planar or axially elongated octahedral coordination. Such differences in the coordination environment of *A*-sites can influence the tendency toward V–V dimer formation.

Our experiments revealed that CuVO_3_ is an insulator and its spins remain fluctuating down to low temperatures. To explain this nature, we hypothesize that a liquid state of spins and V 3d orbitals is realized in CuVO_3_. In the dimerization process, adjacent V ions move closer to each other, resulting in the selection of one of the three 3d orbitals. However, in CuVO_3_, the repetitive formation and dissociation of V–V bonds, namely resonating valence state, may occur in three V–V directions even at low temperatures. Their spins also appear to be in a liquid state. In MgVO_3_, a V–V dimer liquid state occurs at temperatures above the dimerization temperature, which is caused by thermal fluctuations.^3^ To clarify our hypothesis of the V–V dimer liquid state in CuVO_3_, precise spectroscopic experiments will be performed using the high-quality single-crystal samples.

## 4 Conclusion

In this study, we investigated triclinic ilmenite-type CuVO_3_ with Cu^2+^ and V^4+^ honeycomb lattices. The triclinic CuVO_3_ sample was synthesized using high-pressure and high-temperature conditions. The obtained sample exhibited an insulating property and showed no magnetic phase transitions down to 2 K. Crystal structure refinement revealed a Jahn–Teller distortion of CuO_6_ octahedra, resulting in the triclinic structure. In contrast to other ilmenite-type vanadium oxides, which show the V–V dimerization, CuVO_3_ does not exhibit the static dimer state. The Jahn–Teller effect of Cu^2+^ likely competes with V–V dimerization, and the predominance of the former suppresses the latter. A detailed understanding of the orbital state of the V^4+^ ion is an issue to be addressed in future studies. Triclinic ilmenite-type CuVO_3_ would be intriguing as an unusual compound in which different elements adopt distinct orbital states.

## Supplementary Material



## Notes

Synchrotron X-ray powder diffraction patterns of triclinic ilmenite-type CuVO_3_ at 100, 200, 300, 400, 500, 550, and 600 K (Figure S1); temperature dependence of the lattice parameters and unit cell volume of CuVO_3_ (Figure S2); refined structural parameters of CuVO_3_ at 100 K (Table S1); lattice parameters and agreement factors of CuVO_3_ at 100 K (Table S2); refined structural parameters of CuVO_3_ at 300 K (Table S3); lattice parameters and agreement factors of CuVO_3_ at 300 K (Table S4) (PDF)

## Notes

Deposition numbers 2515395 and 2515396 contain the supplementary crystallographic data for this paper. These data can be obtained free of charge via the joint Cambridge Crystallographic Data Centre (CCDC) and Fachinformationszentrum Karlsruhe Access Structures service.

## Abbreviations

SXRD: synchrotron X-ray diffraction
XAS: X-ray absorption spectroscopy
FC: field cooling
ZFC: zero-field cooling
