# Supplementary material for: Spin and Orbital States in Triclinic Ilmenite-Type CuVO3
Source: ACS Omega. 2026 Mar 31;11(14):22093–8. doi: 10.1021/acsomega.5c13303 (PMC13084503; doi:10.1021/acsomega.5c13303)
Supplement: Supplementary file 1 [file ao5c13303_si_001.pdf]

Supporting Information

Spin and orbital states in triclinic ilmenite-type $CuVO_3$

Hajime Yamamoto[1]*, Keigo Ochi[2], Takuya Aoyama[2], Kenji Ishii[3], Daiju Matsumura[4], Takuya Tsuji[4], Kenya Ohgushi[2], and Tadashi Abukawa[5,1]

[1]*Institute of Multidisciplinary Research for Advanced Materials, Tohoku University, 2-1-1 Katahira, Aoba-ku, Sendai, Miyagi 980-8577, Japan.*

[2]*Department of Physics, Graduate School of Science, Tohoku University, 6-3 Aramaki-Aoba, Aoba-ku, Sendai, Miyagi 980-8578, Japan*

[3]*Kansai Institute for Photon Science, National Institutes for Quantum Science and Technology (QST), 1-1-1 Kouto, Sayo-cho, Sayo-gun, Hyogo 679-5198, Japan.*

[4]*Japan Atomic Energy Agency (JAEA), 1-1-1 Kouto, Sayo-cho, Sayo-gun, Hyogo 679-5198, Japan.*

5International Center for Synchrotron Radiation Innovation Smart, Tohoku University, 2-1-1 Katahira, Aoba-ku, Sendai, Miyagi 980-8577, Japan.

Corresponding Author

Hajime Yamamoto: hajime.yamamoto.a2@tohoku.ac.jp

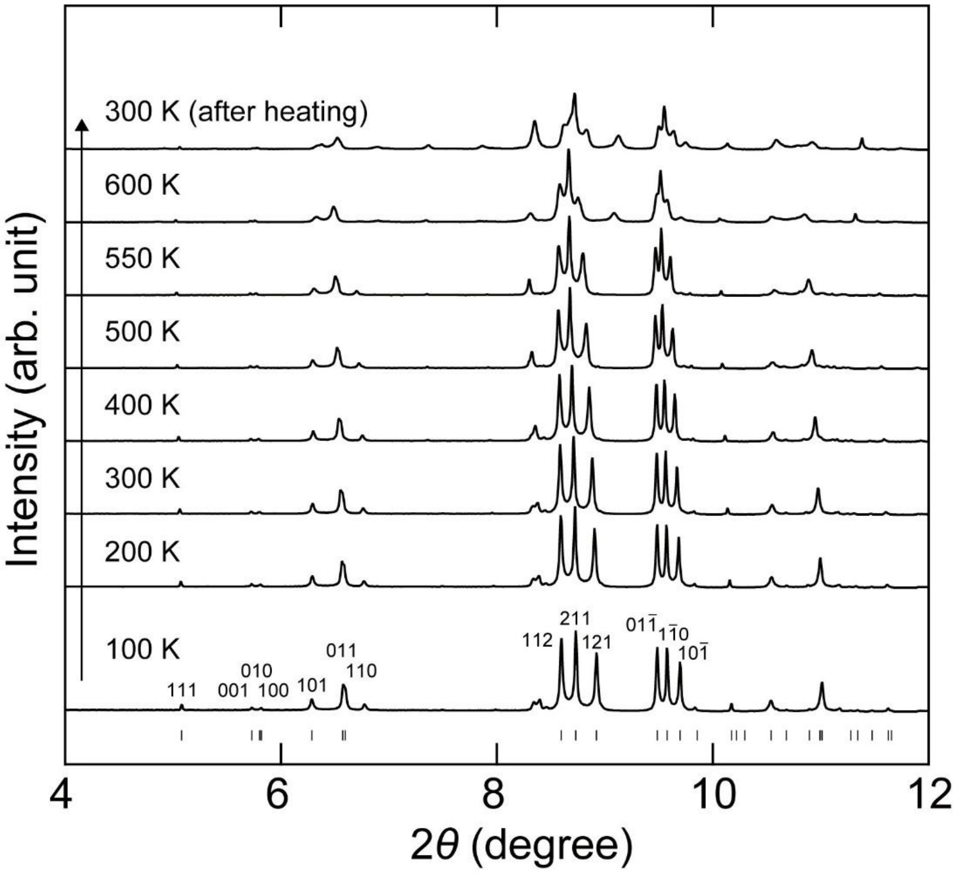

Figure S1. Synchrotron X-ray powder diffraction patterns of triclinic ilmenite-type $CuVO_3$ at 100, 200, 300, 400, 500, 550, and 600 K.

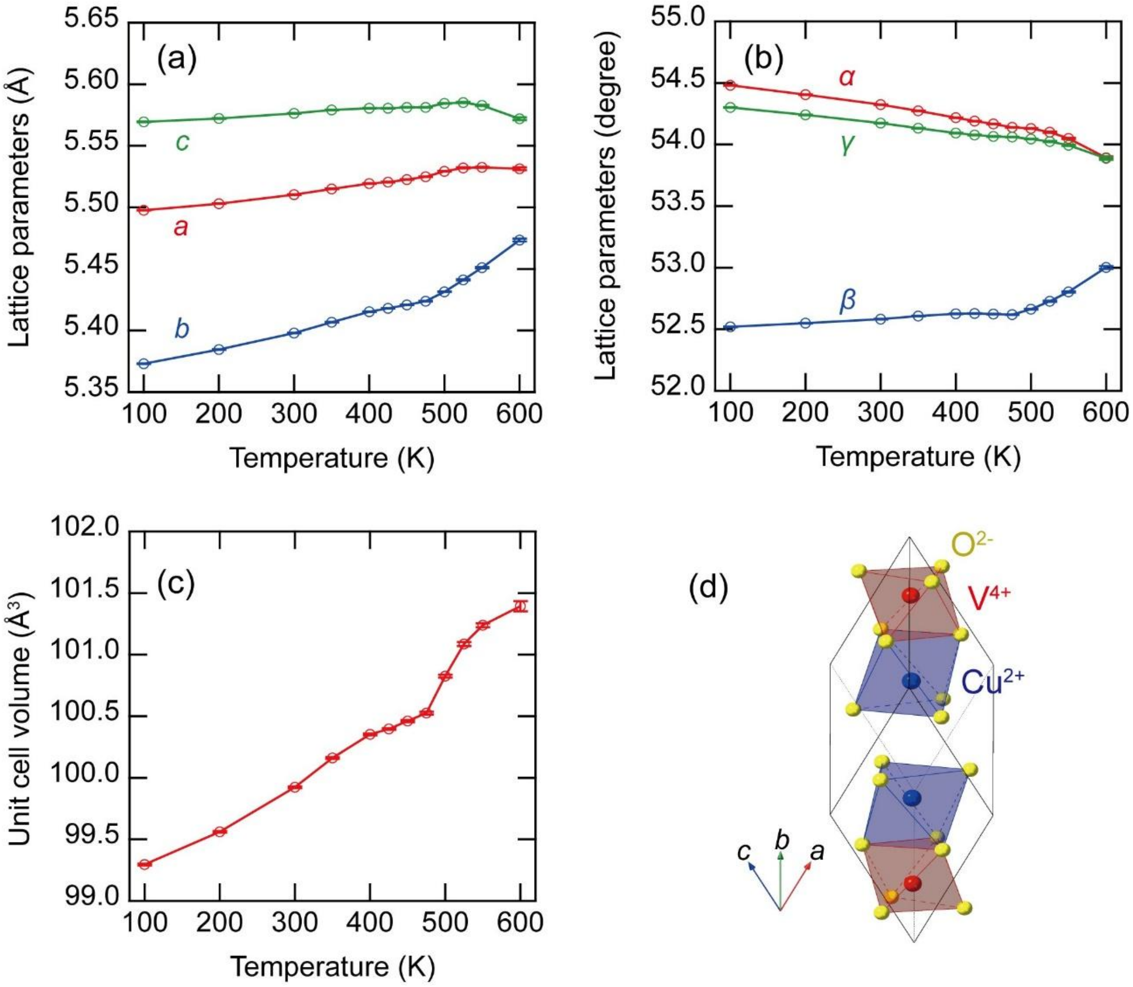

Figure S2. (a) Temperature dependence of the lattice parameters *a*, *b*, and *c*. (b) Temperature dependence of lattice parameters *α*, *β*, and *γ*. (c) Temperature dependence of the unit cell volume. (d) Unit cell of triclinic ilmenite-type $CuVO_3$.

Table S1. Refined structural parameters of $CuVO_3$ at 100 K.

atom	site	occ.	x	y	z	B (Å^2)
Cu	2i	1	0.6390(4)	0.6555(3)	0.6361(3)	0.49(3)
V	2i	1	0.1441(5)	0.1412(5)	0.1512(4)	0.38(3)
O1	2i	1	0.2180(11)	0.5719(11)	0.9313(10)	0.30(5)
O2	2i	1	0.0608(10)	0.7722(12)	0.4418(10)	0.30(5)
O3	2i	1	0.4280(11)	0.0357(11)	0.8022(10)	0.30(5)

The space group was $P\bar{1}$ (No. 2). Z = 2. The temperature factors (B) of O1, O2, and O3 were constrained to be equal.

Table S2. Lattice parameters and agreement factors of $CuVO_3$ at 100 K.

parameter	obtained value
a	5.4978(2)
b	5.3732(2)
c	5.5694(2)
α	54.481(2)
β	52.518(2)
γ	54.300(2)
V	99.296(7)
ρ_{calc}	5.434 g/cm^3
R_{wp}	7.570%
R_B	3.416%

Table S3. Refined structural parameters of $CuVO_3$ at 300 K.

atom	site	occ.	*x*	*y*	*z*	*B* (Å^2)
Cu	2i	1	0.6395(4)	0.6531(4)	0.6359(3)	0.92(3)
V	2i	1	0.1425(6)	0.1423(5)	0.1502(4)	0.55(4)
O1	2i	1	0.2167(11)	0.5698(11)	0.9346(10)	0.45(5)
O2	2i	1	0.0622(10)	0.7721(12)	0.4405(10)	0.45(5)
O3	2i	1	0.4287(10)	0.0379(10)	0.8017(10)	0.45(5)

[a]Space group: $P\bar{1}$ (No. 2). $Z = 2$. The temperature factors (*B*) of O1, O2, and O3 were constrained to be equal.

Table S4. Lattice parameters and agreement factors of $CuVO_3$ at 300 K.

parameter	obtained value
a	5.5103(2)
b	5.3981(2)
c	5.5765(2)
α	54.323(2)
β	52.582(2)
γ	54.173(2)
V	99.922(7)
ρ_{calc}	5.400 g/cm^3
R_{wp}	7.395%
R_B	3.983%
